# Probing the world's largest oceanic plateau: from making to collision

**DOI:** 10.1093/nsr/nwaf176

**Published:** 2025-04-30

**Authors:** Xiaodian Jiang, Zheng-Xiang Li, Wei Gong, Lei Xing, Deyong Li, Hongwei Liu, Chong Xu

**Affiliations:** Ocean University of China, China; Earth Evolution and Dynamics Research Center (EDRC), Laoshan Laboratory, China; Earth Dynamics Research Group (EDRG), School of Earth and Planetary Sciences, Curtin University, Australia; Ocean University of China, China; Ocean University of China, China; Ocean University of China, China; Ocean University of China, China; Ocean University of China, China

## Abstract

The enormously thick Ontong Java Plateau crust was built primarily by deep magma intrusion and underplating, with relatively minor contribution from flood basalts.

Oceanic plateaus represent abnormally thick oceanic crusts linked to mantle plume-induced magmatism [1], but little is known about their detailed anatomy (see Supplementary Materials for a brief review). Here we report high-resolution reflection seismic and ocean bottom seismometer (OBS) data for southwest Ontong Java Plateau (OJP; Fig. 1a) to probe its internal structure and construction history. The data (Fig. 1b, c) reveal that of the total crustal thickness of ∼23 km for the OJP here (Fig. 1b), there is ∼1 km of marine sediments overlaying only ∼3 km of layered basalts (Fig. 1c) formed during two major episodes of plume eruption, likely at ∼116–108 Ma, on top of the original oceanic upper crust. This suggests a 12–15 km of additional plume-induced thickening in the mid- to lower crust through mafic and ultramafic underplating and intrusion (Fig. 1d). In addition, seismic reflection data document the crustal structure of the OJP's collision zone with the Solomon Arc, with young trench-parallel mafic magmatism developed as petit-spot volcanos on the subducting Pacific plate due to lithospheric flexure.

**Figure 1.**
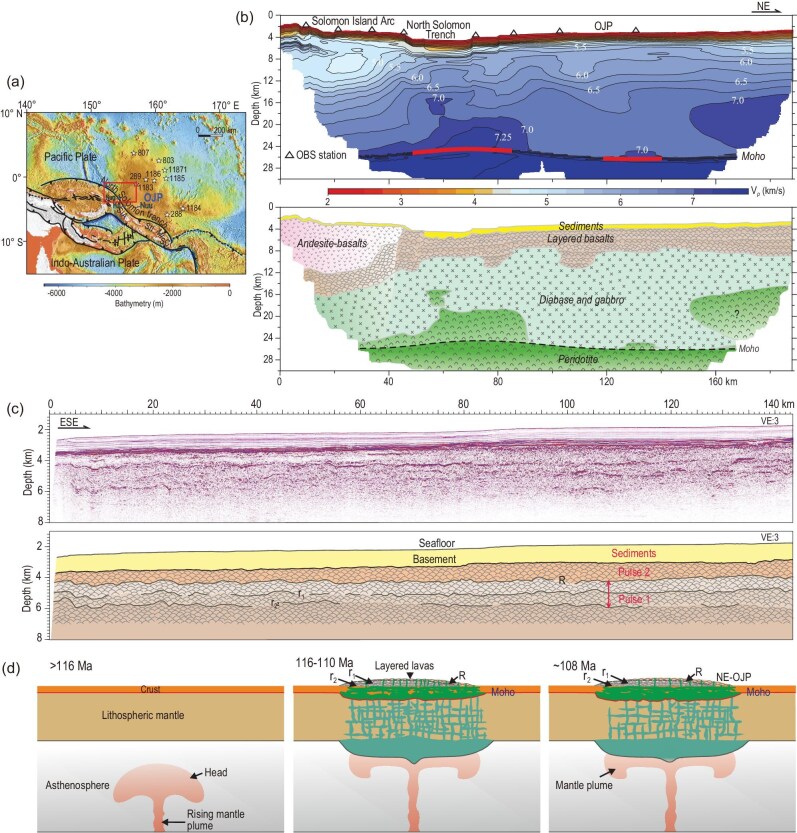
Tectonic framework and bathymetry of the study region (a), OBS tomographic image of traverse L1 as in Fig. S1 and interpreted crustal composition and structure of both the OJP and the colliding Solomon Island Arc (b), seismic traverse L2 (see Fig. S1 for location) in combination with DSDP/ODP drilling results [3] revealing basaltic and sedimentary structure of the OJP (c, with vertical exaggeration (VE) = 3), and schematic diagram showing the structural evolution of the OJP (d). Also shown in (a) locations of the DSDP/ODP drill sites (stars) and other tectonic features (see Fig. S1 for details). In (b), the velocity contour interval is 0.25 km s^−1^ with results for *Pg* and *PmP* phases (see Fig. S11 and Table S2) being used, and lithological interpretation based on *P*-wave velocities. The model in (d) features a thickening of OJP's crust through both basaltic eruptions (layers above reflector r2) and mafic-ultramafic underplating (shown in green), and that of thickening of its mantle lithosphere through mafic-ultramafic intrusions and mantle depletion (shown in blue) above the plume-head. The second episode of flood basalts (shown in orange above reflector R) likely thickens toward the northeastern section of the plateau as documented by recent age determinations [4] (right-hand side of the figure in (d)). Background relief map in (a) is based on GEBCO (General Bathymetric Chart of the Oceans) dataset (https://www.gebco.net/data_and_products/gridded_bathymetry_data/gebco_2021/).

Our multi-channel seismic (MCS) transects provide high resolution images of the upper crust (Fig. 1c and Figs S1–S4) of the OJP. As the two parallel transects of L2 and L3 are only ∼45 km apart (Fig. S1) and show similar structures, here we just describe transect L2 (see transect L3 in Fig. S4). There is a series of sub-horizontal and continuous strong reflectors at the depth of ∼3.5 km with a total thickness of 200–300 m (Fig. 1c and Figs S2–S4), interpreted as the top layers of the basaltic basement of Cretaceous age [2]. The relatively weak but thinly laminated sub-horizontal reflectors above this strong reflecting band thus represent post-eruption, 800–900 m-thick marine sedimentary cover deposited in a calm environment with no obvious tectonic deformation, as observed in central and northern OJP [2].

Lower-frequency but strong sub-horizontal reflectors are observed below the sediment-basalt (basement) interface (Fig. 1c and Figs S1–S4), with the top layer of this package revealed by the >200 m penetration of ODP 1183 and 1185 drilling below the interface to be basalts [2,3]. The data reveal the presence of four packages of sub-horizontal and laterally continuous layered basalts on the top of the plateau within a regional (>140 km) extent, divided by three continuous and strong reflectors (R, r1 and r2; Fig. 1c) with R occurring at the depth of ∼4.4 km (Fig. S5). There is a clear contrast of ∼1 km s^−1^ in *P*-wave velocity and a significant contrast of ∼200 000 gcm^−2^s^−1^ in impedance across R (Figs S5 and S6), indicating a possible difference in the basalt texture. Compared to previous seismic work [2], our new results are of higher resolution, particularly in the depth range of ∼4–6.5 km (or ∼3.8–6 s two-way travel time), and show more clearly the lateral continuity of the major reflectors (Fig. S7) which should be expected of flood basalts on oceanic plateaus.

Recent high-precision ^40^Ar/^39^Ar incremental heating ages determined from lavas penetrated by DSDP 289 and ODP 807, 1183, 1185, 1186 and 1187, and dredged lava samples at OJP (see Fig. 1a for locations), revealed a younger than previously thought age range of ∼116 Ma to ∼108 Ma [4]. The volcanic layers are significantly younger than the regional oceanic crustal age of >130 Ma [5]. In view of the significant difference in their velocity structures (Fig. 1b and Figs S5–S6), we interpret seismic reflector R to represent the bounding surface between two episodes of lava eruptions of the OJP. There are also two discontinuous but traceable weak reflectors, r1 and r2, below R (Fig. 1b), with r1 interpreted as an interlaying boundary between pules of basaltic eruption of the ∼116 Ma episode, and r2 the contact between the original ocean crust basalts and the ∼116 Ma OJP basalts. The interpretation of r2 being the original basaltic seafloor is consistent with the observation that no laterally extensive sub-horizontal reflector, as in strata above it, is found below it (Fig. 1c and Figs S2, S4), as laterally extensive large lava flows should only be expected of eruptions over oceanic plateaus, not in a normal mid-ocean ridge environment.

Figures S2 and S3 show the deformation in the upper crust of the OJP when it approaches the Solomon Island Arc. An increasing number of thrust faults, steepening toward the North Solomon Trench, have been developed in the top 10 km of the OJP crust, likely accompanied by some strike-slip motions with flower-like structures resulting from the oblique convergence [6]. We note that the OJP upper crust, though faulted, retained its overall integrity with a total horizontal structural shortening, estimated through structural section balancing of the transect, of ∼6 km (also see Discussion). On the arc side of the traverse, there are normal faults instead of thrusts, indicating that the elevated forearc region is in a collapse mode.

There are also some mound-like seismic structures defined by discontinuous high-amplitude reflections relative to the surrounding regions in the OJP upper crust near the trench (Figs S2, S3, S8), implying that they do not represent laminated basaltic or sedimentary rocks (Fig. S6). There is no vertical offset in sub-horizontal reflections across the chaotic zones, suggesting that they are not fault reflections. We interpret such sub-vertical chaotic mound-like structures as near-vertical mafic dikes intruding the upper crust.

The crustal velocity image of the OJP–Solomon Island Arc traverse (Fig. 1b) is determined by seismic refraction and reflection travel time data from eight ocean bottom seismometers (OBS) set along traverse L1 (Fig. S1). The low velocities of 2 km s^−1^ ≤ *Vp* ≤ 3.5 km s^−1^ for the top 1 km below the seafloor represent oceanic sedimentary layers, consistent with DSDP/ODP reports [3] and seismic refraction-reflection results [7] (Fig. S3). The ∼3 km-thick layered basalts at the top of the oceanic plateau and the underlaying normal ocean crust basalts, as identified by our MCS results (Fig. 1c and Figs S2–S7), are shown in the OBS transect as having *P*-wave velocities of 5–6 km s^−1^ (Fig. 1b).

The crust-mantle boundary (Moho) is identified at the depth of ∼26 km along the OJP–Solomon Island Arc traverse (Fig. 1b), which is constrained by both crustal refractions (*Pg*) and Moho reflections (*PmP*). This indicates a crustal thickness of ∼23 km for the OJP north of the North Solomon Trench. Here the crust underneath the basaltic layers from a depth of ∼8 km mounts to ∼18 km thick, and exhibits *Vp* of ∼6–7 km s^−1^ (Fig. 1b upper). This mid- to lower plateau crust is interpreted to consist mainly of diabase and gabbro (Fig. 1b lower). The relatively lower velocities ∼7 km s^−1^ immediately below the Moho compared to that of normal ∼120 Ma oceanic lithosphere [8] possibly reflect a combined effect of a somewhat warmer temperature for the thickened lithosphere generated by the impinging hot mantle plumes and associated melts, as well as the likely presence of a network of mafic feeder systems in the mantle lithosphere (see below).

An interesting observation is the presence of lithosphere-like high (>7 km s^−1^) velocity bodies in the thickened lower OJP crust which differ from the ∼15-km thick uniform high-velocity (7.2–7.5 km s^−1^) layer identified in the lower crust of the southern OJP [7]. We interpret such high-velocity regions as ultramafic intrusions (cumulates?) in the crust (Fig. 1b). Under the Solomon Island Arc at the southwestern end of the transect, there is a broad region of up to 8 km-thick in the upper crust under the sedimentary layer, with *P*-wave velocity significantly lower than 5 km s^−1^ (Fig. 1b upper). This region is interpreted to be the andesitic Solomon Island Arc that is underthrust by the OJP.

Figure 1d illustrates the generation and evolution of the OJP, whereas Fig. S9 shows its current collision with the Solomon Island Arc based on our new results. It started with the ∼116 Ma episode of first (and likely major) plume activation in the Pacific Ocean [4]. A large amount of deep mantle-derived magma, centered at 24°S ± 4° in the South Pacific [9], rapidly emplaced over the sea floor of the Pacific plate [5] to form large-scale basaltic layers, thus starting the construction of the OJP [10] (Fig. 1d). At the same time, basaltic underplating and intrusions, with occasional intrusion or underplating of ultramafic rocks, started to thicken the entire oceanic crust. A network of melt feeders, as well as newly depleted mantle generated by the plume-induced high-degree melting, also thickened the oceanic mantle lithosphere [11] (Fig. 1d). A second episode of similar processes likely occurred over much of the OJP, including the studied region, prior to ∼110 Ma, as documented by the widespread ∼116–110 Ma basaltic age distribution over the plateau [4], generating the two pulses of basaltic eruptions in the studied region (Fig. 1b and d). This latter episode may have lasted to as young as ∼108 Ma, mostly along the northeastern section of the plateau, as documented by recent age determinations [4] (Fig. 1d, 108 Ma).

We illustrate in Fig. S9 a cartoon sketch of the collision of the OJP with the Solomon Island Arc, featuring the jam of subduction by the OJP possibly soon after the collision at 25–20 Ma [12]. Assuming only the lower OJP crust has been subducted under the Solomon Island Arc [13], the estimated ∼6 km horizontal shortening deformation of the oceanic upper crust in traverse L1, as shown in Fig. S3, is taken as the maximum subduction of the OJP beneath the island arc. This suggests an average subduction rate of the oceanic plateau at a very low value of ∼0.2 mm/yr.

The presence of a high velocity body with *Vp* ≥7 km s^−1^ above the Moho in the region beneath the Northern Solomon Trench (Fig. 1b) suggests the possible underplating of mafic-ultramafic magmatism above the Moho. Such magma likely formed during the convergence between the OJP and the Solomon Island Arc where extension occurs at basal lithosphere either by lithospheric flexure when the oceanic plate approaches the trench [12,14], and/or due to upward rebound of the lithosphere after the break-off of the subducted oceanic slab [15] (Fig. S9). The magma chamber above the Moho, as in Fig. 1b and Fig. S9, likely led to the mafic intrusions as interpreted in the MCS transect (Figs S3 and S8) and the occurrence of young, trench-parallel volcanos (petit-spot volcanos [14]) north of the North Solomon Trench (e.g. the Nuugurigia, Kilinailau and Nuguria islands) (Fig. S9). A correlation of drilling results of DSDP 289 and ODP 1183 with MCS data shows that the ooze to chalk unit cut by the interpreted intrusions has a deposition age of middle Miocene (∼16–10.5 Ma) [3], suggesting a young magma emplacement time of syn- to post ∼16–10.5 Ma. We note that Hanyu *et al.* [12] preferred the simple near-trench lithospheric flexure for the formation of the near-trench mafic intrusions like those near the Japan Trench [12,14].

## Supplementary Material

nwaf176_Supplemental_File

## DATA AND MATERIALS AVAILABILITY

Original MCS and OBS data are available at [Zenodo link doi:10.5281/zenodo.10944507].
