# Supplementary material for: Probing the world's largest oceanic plateau: from making to collision
Source: Natl Sci Rev. 2025 Apr 30;12(7):nwaf176. doi: 10.1093/nsr/nwaf176 (PMC12201981; doi:10.1093/nsr/nwaf176)
Supplement: nwaf176_Supplemental_File [file nwaf176_supplemental_file.pdf]

Supplementary Materials for

Probing the world's largest oceanic plateau: from making to collision

Xiaodian Jiang[1], Zheng-Xiang Li[2,3]*, Wei Gong[1], Lei Xing[1], Deyong Li[1], Hongwei Liu[1], Chong Xu[1]

[1] Ocean University of China, 238 Songling Road, Qingdao, China
[2] Earth Evolution and Dynamics Research Center (EDRC), Laoshan Laboratory, Qingdao, Postcode 266237, China
[3] Earth Dynamics Research Group (EDRG), School of Earth and Planetary Sciences, Curtin University, GPO Box U1987, Perth, Western Australia 6845, Australia

*Corresponding author. Email: Z.Li@curtin.edu.au

This PDF file includes the following content: **Page**

- A brief literature review on the Ontong Java Plateau (OJP) and its collision with the Solomon Island Arc 2
- Data acquisition 3
- Fig. S1. Tectonic framework and bathymetry of the study region showing the locations of the geophysical survey traverses 4
- Fig. S2. Interpretation of seismic reflection results along traverse L1 from the Solomon Island Arc to the OJP 5
- Fig. S3. Zoomed-in seismic reflection data and geotectonic interpretation along traverse L1 emphasizing the collision of the OJP with the Solomon Island Arc 5
- Fig. S4. Basalt layered structural cross section of the OJP for traverse L3 based on interpretation of seismic reflection results and DSDP/ODP drilling data 6
- Fig. S5. One-dimensional P-wave velocity structure of the upper crust of the OJP inverted using the Jason program, a petroleum seismic exploration software 6
- Fig. S6. P-wave impedance of layered basaltic structures of the OJP for seismic traverse L2 7
- Fig. S7. Correlation of ODP drilling results (Leg 192) and previous seismic data with multichannel seismic reflection data of this study (L2) 7
- Fig. S8. Zoomed-in seismic reflection data and geotectonic interpretation along traverse L1 emphasizing possible mafic intrusions 8
- Fig. S9. Tectonic model of magmatic activity induced by rapid rebound of the front end of the OJP caused by slab break-off 8
- Fig. S10. MCS data processing workflow 9
- Fig. S11. Two P-wave phases, Pg and PmP, as picked in the OBS seismic record sections 9
- Fig. S12. Travel-time plot of seismic phases (colored by phase) picked at each OBS station 10
- Table S1. Key parameters used for MCS data processing 10
- Table S2. Misfits between travel time picks and model predictions 10
- References 11

A brief literature review on the Ontong Java Plateau (OJP) and its collision with the Solomon Island Arc

Oceanic plateaus are the manifestation of mantle plumes from the deep Earth [1], and may have played a critical role in the formation of continental crust through obduction [2]. However, as they are mostly submerged in the deep oceans, little is known about their internal structures beyond the top few kilometers, their construction histories, and their behavior at convergent plate margins. The Ontong Java Plateau (OJP; Fig. S1 inset) is a massive submerged smooth platform with an elevation of ca. 2,000 m above the surrounding seafloor in the western Pacific. It is generally believed to be a large igneous province induced by deep mantle plumes [3-5], although mechanisms such as passive upwelling of mantle near a rapidly expanding ridge [6-7] and massive melting of mantle initiated by a major bolide impact [8] have also been proposed. The estimated total crustal volume of the plateau is 44.4×10^6 km^3 [6], making it the largest oceanic plateau preserved in the world. Therefore, the OJP provides an ideal window for studying the crustal building process, including plateau structure and evolution, and related large-scale plume magmatism in an oceanic environment [3, 9-10]. The broader region, including the Solomon Island Arc and the OJP, also provides an excellent opportunity for analyzing the dynamic characteristics of collision process between an oceanic plateau and a magmatic arc in a convergent plate boundary.

Although early seismic refraction surveys revealed an anomalously thick oceanic crust of ~25–42 km for the OJP [11], up to ~five times of that of normal oceanic crust, the internal architecture and thus the thickening mechanism of such enormously thick oceanic crust remains elusive. Similar crustal thicknesses were revealed by more recent Rayleigh waveform tomographic [12] and receiver function [13] analyses.

DSDP/ODP ocean drilling had 13 sites revealing the presence of ca. 1,000 m of thick foraminifera soft mud and limestone over the basaltic "basement" of the OJP, with ODP site 1185 penetrating a further ~217 m of interbedded and non-uniformly distributed pillow lava and massive basaltic flows with single layer thickness ranging between several meters to nearly 100 meters [14-18]. Geochemical analyses of ODP basaltic samples and basalts of obducted slices of the OJP on island arcs revealed relatively monotonous chemical compositions across the plateau, with minor variations allowing them to be grouped into four types: the Kroenke-, Kwaimbaita-, Wairahito-, and Singgalo-type basalts [4, 19]. However, the lack of stratigraphic and precise age constraints on these basaltic types made it difficult to link them with plateau stratigraphy.

Multichannel seismic reflection data also showed the presence of numerous strong reflectors in the top section of the basement, interpreted to be the interbedded structure of pillow lava and massive flood basalt flows with or without sedimentary interbeds [20]. However, with their moderate receiver cable lengths of 600–1200 m and 25 m group interval solid streamer, those workers were only able to image the top 3,500 m of the crust at a lower resolution than that of modern approaches.

What happens when such a massive oceanic plateau reaches the subduction zone is another contentious issue. Most plate reconstructions suggest that the OJP reached the subduction zone under the Solomon Island Arc at 25–20 Ma [21-22], and started a hard-docking stage ~10 Myr later in the Late Miocene-Pliocene [23-25], causing the subduction initiation in the Solomon Sea back-arc basin beneath the Solomon Island Arc with an opposite polarity [23, 26]. Based on analyses of seismic reflection, ocean bottom

seismometer, and other geophysical and geological data, previous studies [23, 27] concluded that about 80% of the OJP crustal thickness is subducting under the Solomon Islands, whereas only the top ~7 km of the supercrustal basaltic and sedimentary rocks are preserved on the overriding plate by subduction–accretion processes. It was further argued that about 50% of the OJP may have already been subducted into the lower mantle [28]. On the other hand, *P*-wave tomography and shear wave analysis revealed that the OJP has a rheologically strong upper mantle root with a chemical or mineralogical heterogeneity [12, 29-30], similar to that of thick-rooted continental cratons, and is therefore expected to ultimately accrete against the island arc as a significant component of future continental growth [2]. Numerical modeling results and buoyancy analysis also suggest that oceanic plateaus such as the OJP are difficult to subduct [31-32]; instead, they tend to jam the subduction as exemplified by the Caribbean Plateau at ~73 Ma [33].

Data acquisition

We report higher-resolution multi-channel seismic reflection and ocean bottom seismometers (OBS) data collected during January/March 2021 aboard the R/V Kexue, with traverses covering the area between western Ontong Java Plateau (OJP) and the Solomon Island Arc across the North Solomon Trench (Figs. 1 and S1). Our results cover the entire crustal thickness of western OJP, the North Solomon Trench, and the Solomon Island Arc. We achieved higher resolution and deep crustal MCS signal record length of 12s by having a longer (2100 m) seismic reflection receiver cable, and acquired higher-resolution OBS results by using a large volume (8000 in^3) air-gun.

Our data were collected along a 187 km SW-NE trending transect (L1) and two nearly W-E trending transects of 146 km and 180 km lengths (L2 and L3; Fig. S1). The MCS data are collected using a NTRS2 offshore multi-channel seismograph system with 3-km-long seismic streamers and a 1680 cu. in. tuned air gun array. Shot intervals were 25 m and the sampling rate was 2 ms. The MCS Data were subjected to conventional seismic data processing methods using the Omega software, including a pre-stack time migration and broadband processing. Anisotropic ray-based Kirchhoff migration was used to migrate the seismic data from the time (ms) domain to the depth (m) domain, constrained by the interval velocity model. Data processing encountered problems such as strong seafloor multiples, great variations in water depth, and large energy differences between seismic traces. The workflow used for MCS data processing is described in Figure S10, with Table S1 providing further details of the MCS data processing parameters used.

Along transect L1, eight short-period OBS stations with an active source were deployed. OBS contained a hydrophone and a 3-component geophone that recorded at a sampling rate of 50 Hz. The active source was a 4-string, 8000-cubic-inch air-gun array, towed at 10–20 m depth with a shot space of ~180 m at a speed of 4–5 knots. The recorded data were converted to SEG-Y format and time-corrected for clock drift. Water wave travel times were used to locate the instruments on the seafloor. Instrument locations were determined from travel times of the direct water wave using a Bayesian grid-search algorithm [34]. The processing of the active source OBS data consists of time correction, instruments relocation [34], 5–24 Hz minimum-phase Butterworth filter, range-dependent amplitude gain, predictive deconvolution and static correction. Two P-wave phases, Pg and PmP, were identified in the record sections (Fig. S11). Travel-time plot of seismic phases picked at each OBS station are shown in Figure S12. A nonlinear joint refraction and reflection travel time tomographic technique [35] was used to determine the crustal P-wave velocity structure and the geometry of the crust-mantle boundary.

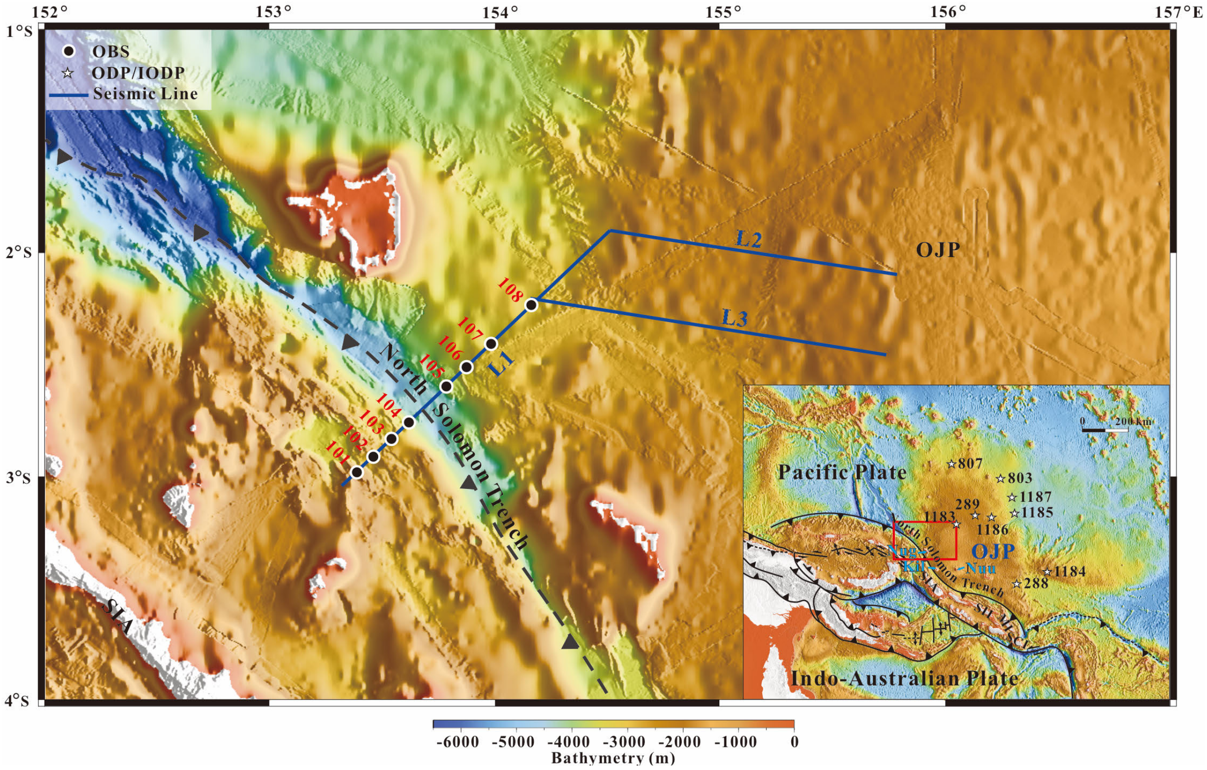

Fig. S1. Tectonic framework and bathymetry of the study region showing the locations of the geophysical survey traverses. Blue lines L1-L3 mark the location of three seismic reflection survey traverses depicted in Figure 1c and Figures S2–S4. Also shown are positions of ocean bottom seismometers (OBSs 101–108; black dots) with active source, with results depicted in Figure 1b, and locations of the DSDP/ODP drill sites (stars). ODP 1183, 1185 and 1186 sites drilling data revealed an ooze-chalk-limestone composition for the sedimentary cover, representing undisturbed pelagic sedimentation, and a slow deposition rate of ≤0.01 mm/yr (calculated after Ref. 18). Background relief map is based on GEBCO (General Bathymetric Chart of the Oceans) data set (https://www.gebco.net/data_and_products/gridded_bathymetry_data/gebco_2021/). SIA, Solomon Island Arc; SII, Santa Isabel Island; MI, Malaita Island; SCI, San Cristobal Island; Nug, Nuguria; Kil, Kilinailau; Nuu, Nuugurigia.

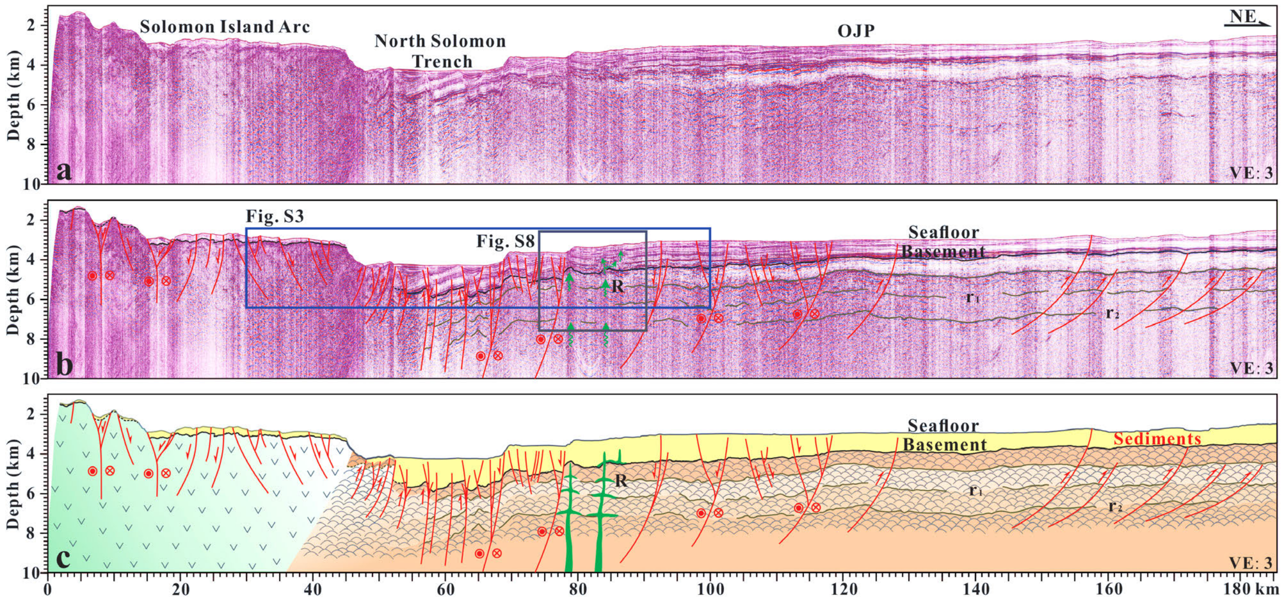

Fig. S2. Interpretation of seismic reflection results along traverse L1 from the Solomon Island Arc to the OJP. (a) Pre-stack time-migrated seismic profile. (b) Structural interpretation. (c) Overall geological interpretation including possible mafic intrusions (shown in green). Vertical exaggeration (VE) = 3.

-

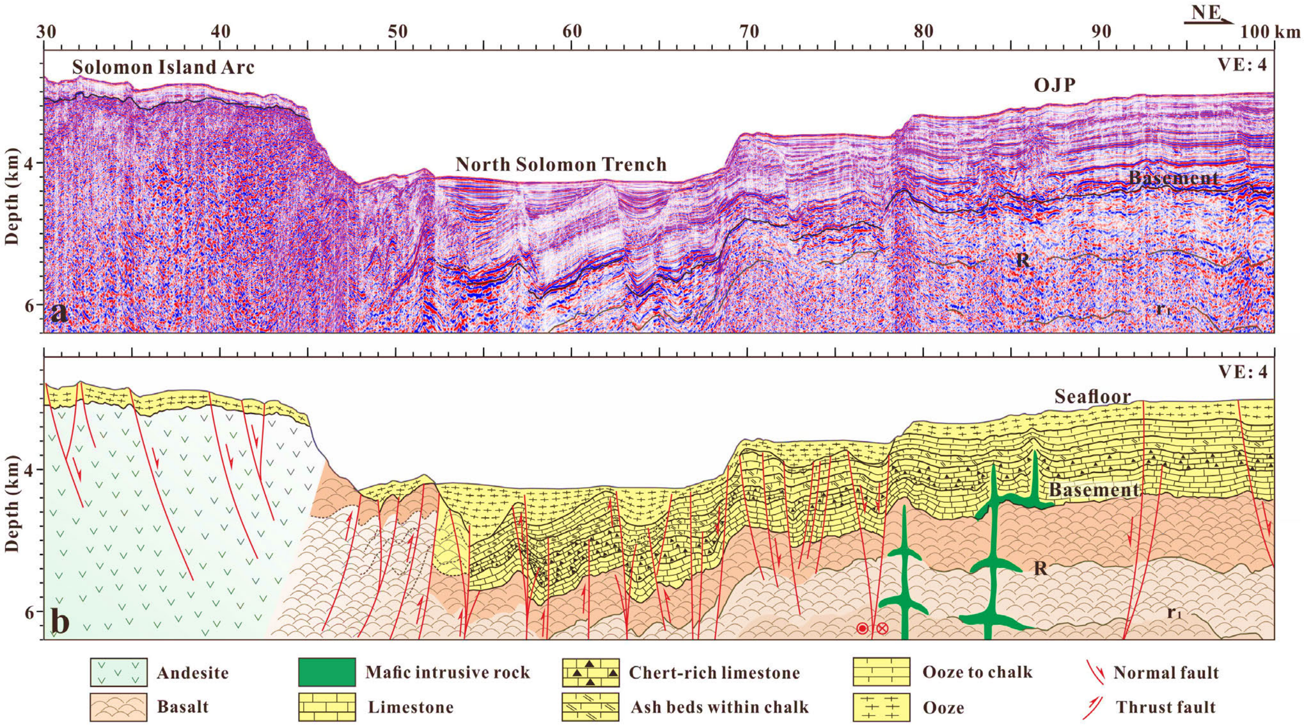

Fig. S3. Zoomed-in seismic reflection data and geotectonic interpretation along traverse L1 emphasizing the collision of the OJP with the Solomon Island Arc (for full L1 traverse see Fig. S1). (**a**), High-resolution seismic image of the top layers of OJP and the Solomon Island Arc which collide along the North Solomon Trench. (**b**), Detailed geotectonic interpretation of collision zone across the North Solomon Trench. The ocean sedimentary lithostratigraphy is based on DSDP/ODP reports [18] and previous studies [24]. Vertical exaggeration (VE) = 4.

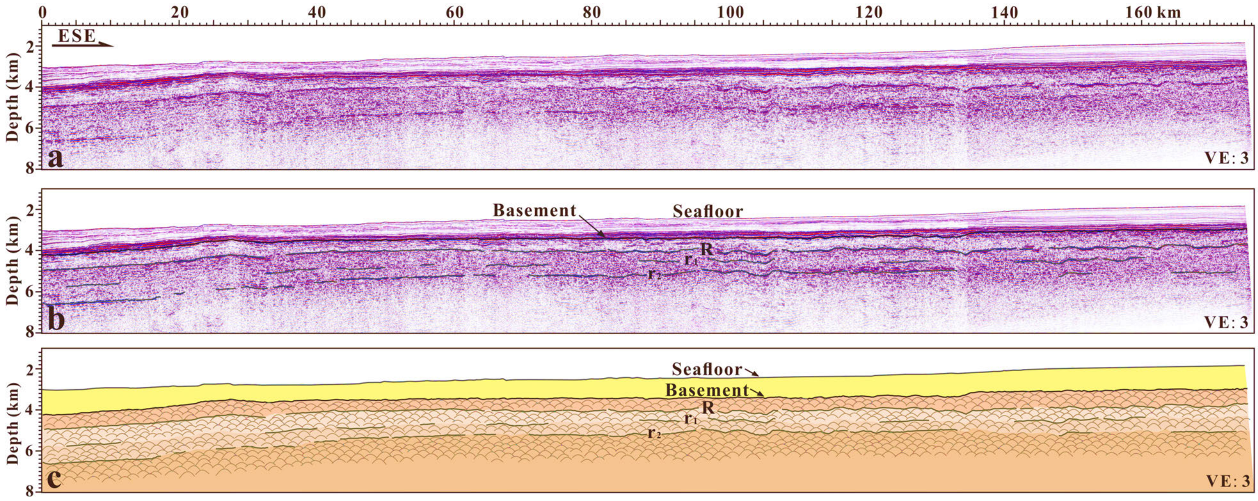

Fig. S4. Basalt layered structural cross section of the OJP for traverse L3 based on interpretation of seismic reflection results and DSDP/ODP drilling data [17-18]. (a) Pre-stack time-migrated seismic reflection images along the traverse L3; (b) Seismogeological interface tracking based on seismic attributes; (c) Stratigraphic interpretation. Black lines mark the major boundaries. Vertical exaggeration (VE) = 3.

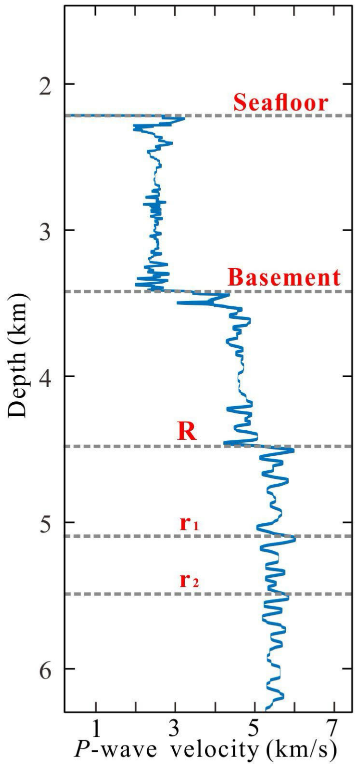

Fig. S5. One-dimensional *P*-wave velocity structure of the upper crust of the OJP inverted using the Jason program, a petroleum seismic exploration software. Based on the trend-constrained sparse spike deconvolution algorithm, the *P*-wave impedance is obtained by finding reflection coefficient which minimizes the objective function. The *P*-wave impedance value was extracted at about x = 59 km on traverse L2, and the relationship between velocity and density follows the Gardner empirical formula, which allows the velocity curve at this location to be determined.

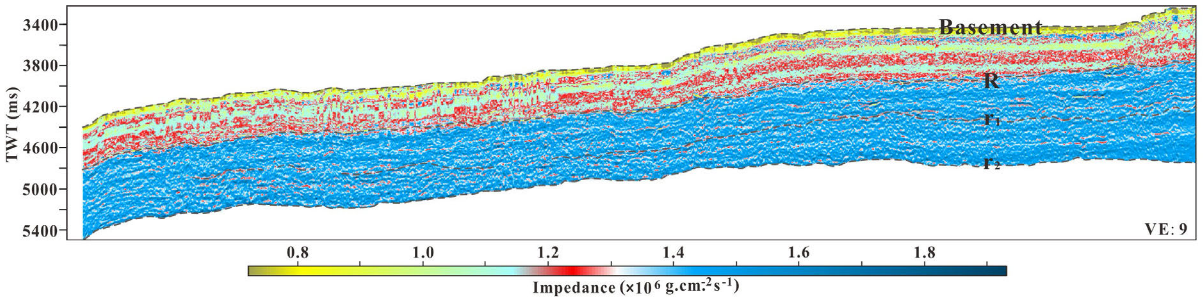

Fig. S6. *P*-wave impedance of layered basaltic structures of the OJP for seismic traverse L2. The *P*-wave impedance was obtained by sparse-spike inversion of post-stack seismic data. Vertical exaggeration (VE) = 9.

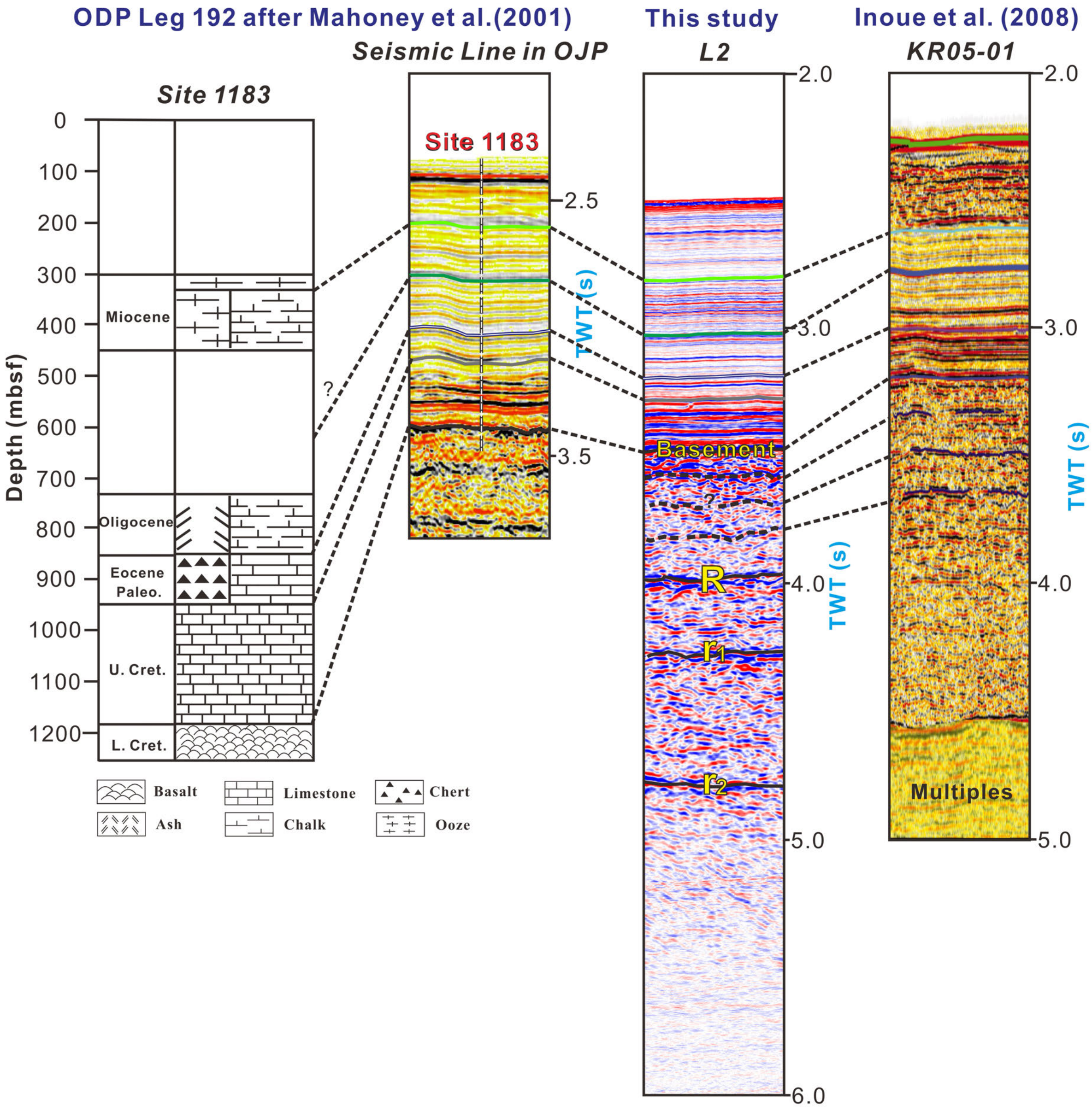

Fig. S7. Correlation of ODP drilling results (Leg 192) and previous seismic data [20] with multichannel seismic reflection data of this study (L2).

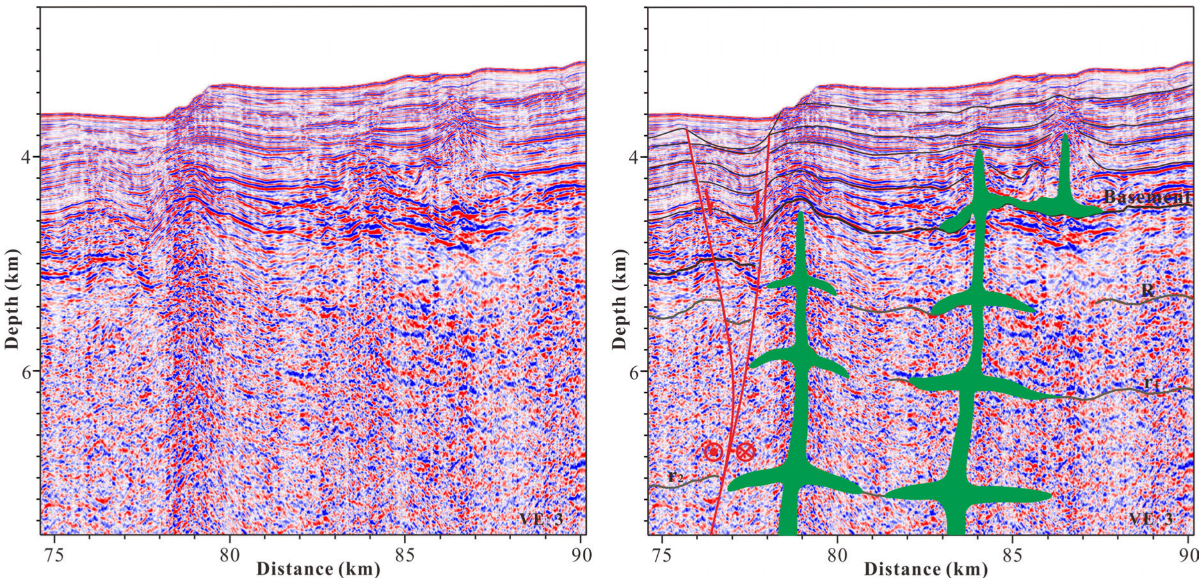

Fig. S8. Zoomed-in seismic reflection data and geotectonic interpretation along traverse L1 emphasizing possible mafic intrusions (shown in green) (for full L1 traverse see Fig. S2). Vertical exaggeration (VE) = 3.

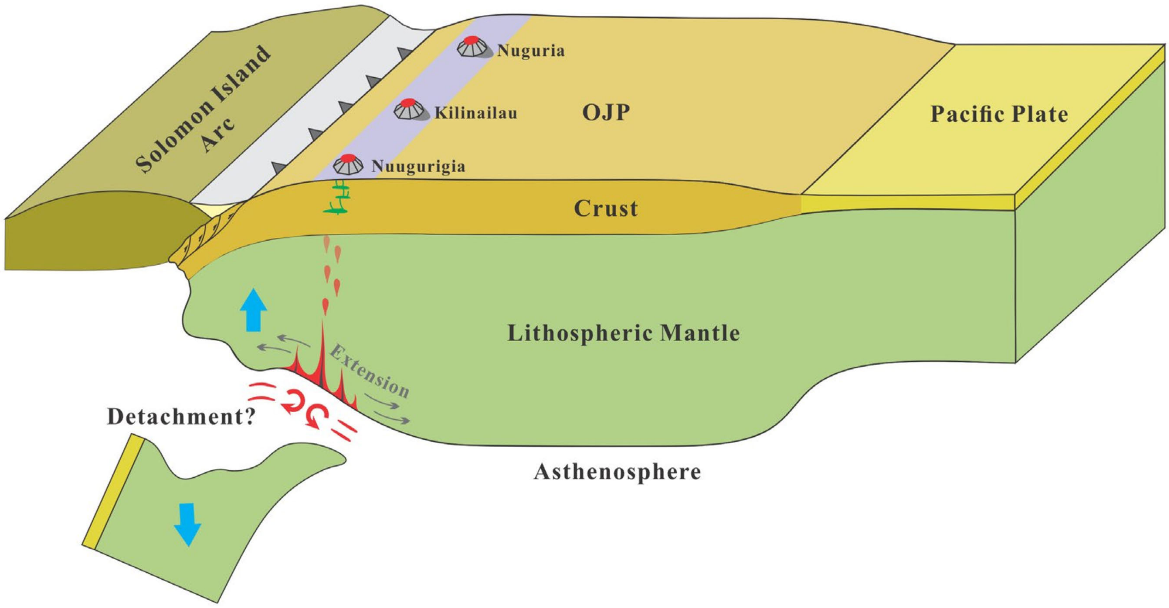

Fig. S9. Tectonic model of magmatic activity induced by rapid rebound of the front end of the OJP caused by slab break-off. This happened after the subduction was jammed by the collision of the OJP (modified after Hanyu et al. [22]). See main text for alternative interpretations.

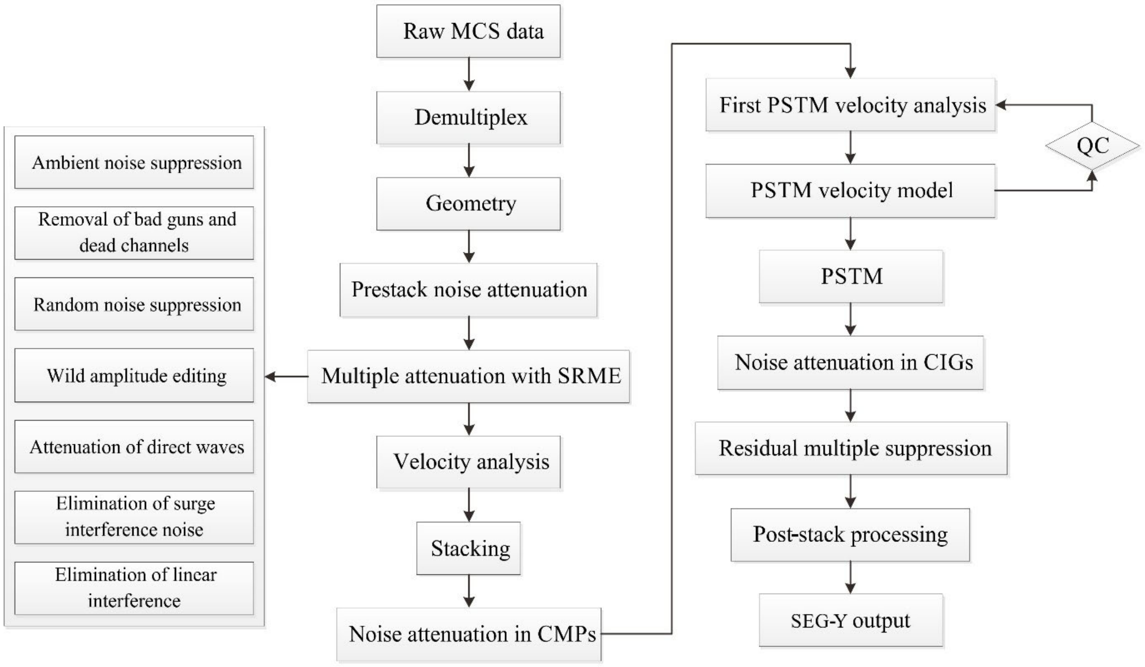

Fig. S10. MCS data processing workflow. For parameters used see Table S1 below.

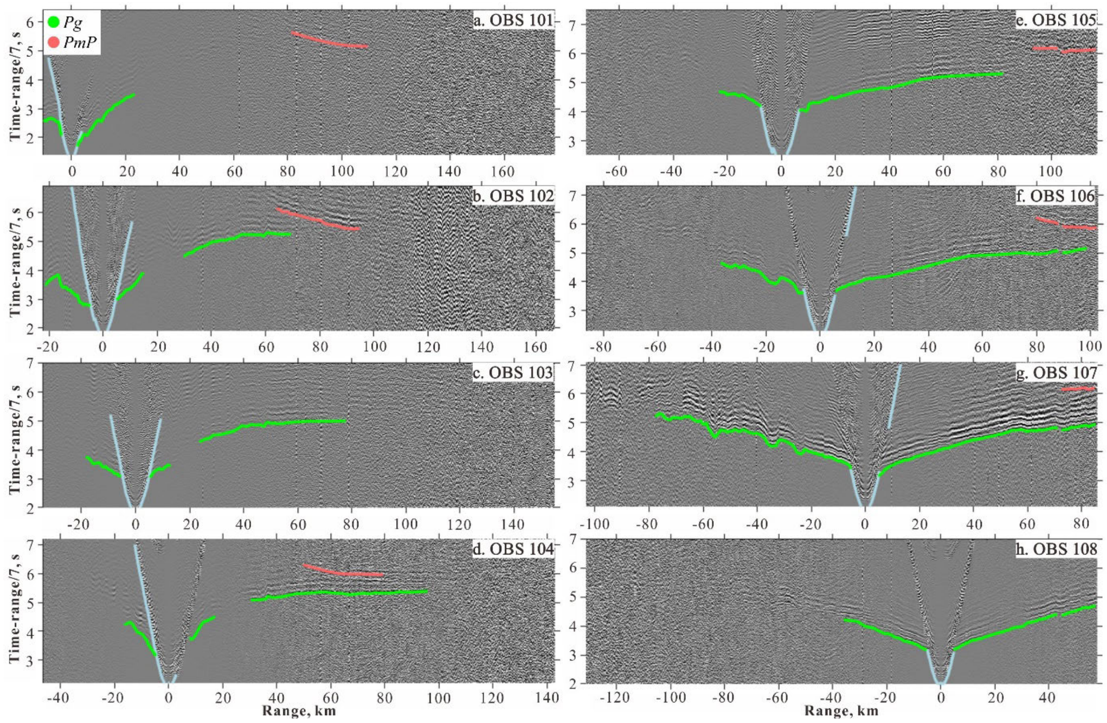

Fig. S11. Two *P*-wave phases, *Pg* and *PmP*, as picked in the OBS seismic record sections (shown by green and red colors, respectively). The seismic data have been band-pass filtered, corrected for seafloor bathymetry and scaled by range. *P*-wave phases of the *Pg* and *PmP* were identified in the record sections, and were picked with pick uncertainties of ± 10~40 ms and ± 70~100 ms, respectively. The *Pg* phase is the refraction travelling through the sediment and crust, with apparent velocities of about 2~7 km/s. The high-amplitude *PmP* phase is the wide-angle reflection from the crust-mantle boundary. Light blue lines indicate direct water wave phase.

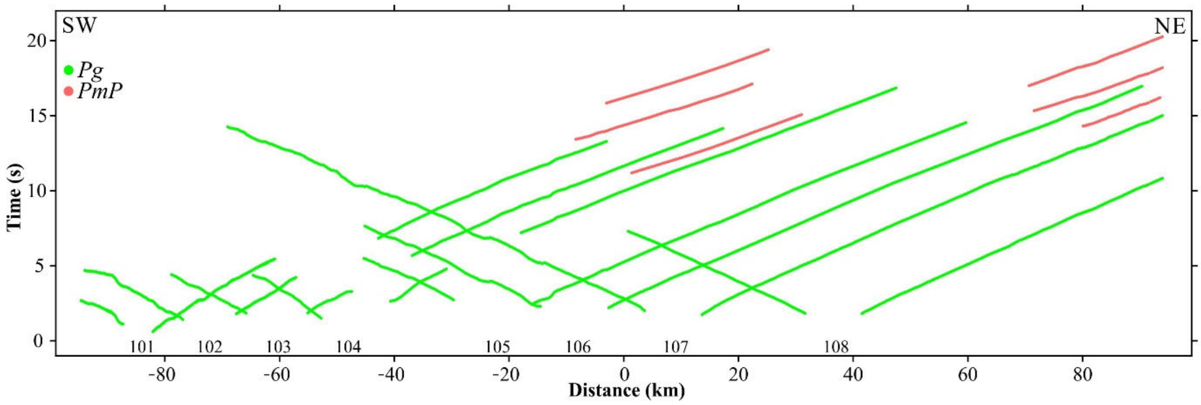

Fig. S12. Travel-time plot of seismic phases (colored by phase) picked at each OBS station. There were 4574 travel times used in the tomographic analysis. Travel times are plotted with distance based on the stations position being the origin for each set of curves. Left trending phases represent shots fired from the southwest of the station and right trending phases represent shots from the northeast.

Table S1. Key parameters used for MCS data processing

Key processing step	Parameters used
Observation system definition	6.25-m bins
Velocity analysis	Each 160 CMPs (1km)
Ambient noise attenuation	5HZ/31DB; Gated times: 51trace/200ms
Multiple attenuation	SRME; Gated times: 100trace/200ms
First PSTM velocity analysis	Each 160 CMPs (1km)
PSTM velocity model updating	Each 160 CMPs (500m)
Residual multiple suppression	High-precision radon transformation (DTCUT180)

Table S2. Misfits between travel time picks and model predictions*

	Pg	***PmP***	**Total**
**Number of picks**	3839	735	4574
**Final mean misfit, ms**	11.31	-11.92	7.58
**RMS misfit, ms**	35.88	32.24	36.33
χ^2	0.88	0.53	0.82

*The velocity model consists of three layers: water column, sediments and crust. For the forward problem, the grid spacing was 250 m laterally and 100 m vertically. For the inverse problem, the perturbation grid spacing was 2–4 km laterally and increased vertically from 0.2 km at the top to 2 km at the bottom vertically. Priori uncertainties were applied to the model slowness and interface depth that act as damping terms to model perturbations, and spatial smoothness constraints were included to improve the solution stability and avoid statistical overfitting. The starting model was built based on the 1-D velocity model of the Pacific Ocean basin [36]. The final velocity model has an RMS misfit of 36.33 ms and a chi-squared misfit of 0.82.

References

1. Koppers AAP, Becker TW, and Jackson MG *et al.* Mantle plumes and their role in Earth processes. *Nat Rev Earth Env* 2021; **2**: 382−401.
2. Ben-Avraham Z, Nur A and Jones D *et al.* Continental accretion: From oceanic plateaus to allochthonous terranes. *Science* 1981; **213**: 47−54.
3. Ishikawa A, Pearson DG and Dale CW. Ancient Os isotope signatures from the Ontong Java Plateau lithosphere: Tracing lithospheric accretion history. *Earth Planet. Sci. Lett.* 2011; **301**: 159−170.
4. Tejada M, Suzuki K and Hanyu T *et al.* Cryptic lower crustal signature in the source of the Ontong Java Plateau revealed by Os and Hf isotopes. *Earth Planet. Sci. Lett.* 2013; **377-378**: 84−96.
5. Chen S-S, Liu J-Q and Gao R *et al.* Geochemistry of Cretaceous basalts from the Ontong Java Plateau: Implications for the off-axis plume−ridge interaction. *Chem. Geol.* 2021; **564**: 119815.
6. Gladczenko TP, Coffin MF and Eldholm O. Crustal structure of the Ontong Java Plateau: Modeling of new gravity and existing seismic data. *J. Geophys. Res. Solid Earth* 1997; **102**: 22711−22729.
7. Korenaga J. Why did not the Ontong Java Plateau form subaerially? *Earth Planet. Sci. Lett.* 2005; **234**: 385−399.
8. Ingle S and Coffin MF. Impact origin for the greater Ontong Java Plateau? *Earth Planet. Sci. Lett.* 2004; **218**: 123−134.
9. Fitton JG and Godard M. Origin and evolution of magmas on the Ontong Java Plateau. *Geo. Soc. Lond. Spec. Pub.* 2004; **229**: 151−178.
10. Covellone BM, Savage B and Shen Y. Seismic wave speed structure of the Ontong Java Plateau. *Earth Planet. Sci. Lett.* 2015; **420**: 140−150.
11. Furumoto AS, Webb JP and Odegard ME *et al.* Seismic studies on the Ontong Java Plateau, 1970. *Tectonophysics* 1976; **34**: 71−90.
12. Richardson WP, Okal EA and Lee S. Rayleigh-wave tomography of the Ontong-Java Plateau. *Phys. Earth Planet. Int.* 2000; **118**: 29−51.
13. Tonegawa T, Miura S and Ishikawa A *et al.* Characterization of crustal and uppermost-mantle seismic discontinuities in the Ontong Java Plateau. *J. Geophys. Res. Solid Earth* 2019; **124**: 7155−7170.
14. Winterer EL, Riedel WR and Brönnimann P *et al.* Initial Reports of the Deep Sea Drilling Project, 1971; **7**.
15. Andrews JE, Packham G and Eade JV *et al.* Initial Reports of the Deep Sea Drilling Project, 1975; **30**.
16. Moberly R, Schlanger SO and Baltuck M *et al.* Initial Reports of the Deep Sea Drilling Project, 1986; **89**.
17. Berger WH, Kroenke LW and Mayer LA *et al.* Proceedings of the Ocean Drilling Program, Scientific Results, 1993; **130**.
18. Mahoney JJ, Fitton JG and Wallace PJ *et al.* Proceedings of the Ocean Drilling Program, Initial Reports, 2001; **192**.
19. Neal CR, Coffin MF and Sager WW. Contributions of scientific ocean drilling to understanding the emplacement of submarine large igneous provinces and their effects on the environment. *Oceanography* 2019; **32**: 176−192.
20. Inoue H, Coffin MF and Nakamura Y *et al.* Intrabasement reflections of the Ontong Java Plateau: Implications for plateau construction. *Geochem. Geophys. Geosyst.* 2008; **9**: Q04014.
21. Zahirovic S, Matthews KJ and Flament N *et al.* Tectonic evolution and deep mantle structure of the eastern Tethys since the latest Jurassic. *Earth-Sci. Rev.* 2016; **162**: 293−337.

22. Hanyu T, Tejada MLG and Shimizu K *et al.* Collision-induced post-plateau volcanism: Evidence from a seamount on Ontong Java Plateau. *Lithos* 2017; **294–295**: 87–96.
23. Mann P and Taira A. Global tectonic significance of the Solomon Islands and Ontong Java Plateau convergent zone. *Tectonophysics* 2004; **389**: 137–190.
24. Miura S, Suyehiro K and Shinohara M *et al.* Seismological structure and implications of collision between the Ontong Java Plateau and Solomon Island Arc from ocean bottom seismometer–airgun data. *Tectonophysics* 2004; **389**: 191–220.
25. Taira A, Mann P and Rahardiawan R. Incipient subduction of the Ontong Java Plateau along the North Solomon trench. *Tectonophysics* 2004; **389**: 247–266.
26. Petterson MG, Babbs T and Neal CR *et al.* Geological–tectonic framework of Solomon Islands, SW Pacific: crustal accretion and growth within an intra-oceanic setting. *Tectonophysics* 1999; **301**: 35–60.
27. Phinney EJ, Mann P and Coffin MF *et al.* Sequence stratigraphy, structural style, and age of deformation of the Malaita accretionary prism (Solomon arc-Ontong Java Plateau convergent zone). *Tectonophysics* 2004; **389**: 221–246.
28. Maruyama S, Utsunomiya A and Ishikawa A. Ontong-Java Plateau, the world's largest oceanic plateau, has been subducted 50%, with the remaining 50% on the surface, and with a < 1% accretion on the hanging wall of the Solomon islands. *J. Geogr*. 2011; **120**: 1035–1044.
29. Klosko ER, Russo RM and Okal EA *et al.* Evidence for a rheologically strong chemical mantle root beneath the Ontong–Java Plateau. *Earth Planet. Sci. Lett.* 2001; **186**: 347–361.
30. Gomer BM and Okal EA. Multiple-ScS probing of the Ontong-Java Plateau. *Phys. Earth Planet. In.* 2003; **138**: 317–331.
31. Sun B, Kaus BJP and Yang J *et al.* Subduction Polarity Reversal Triggered by Oceanic Plateau Accretion: Implications for Induced Subduction Initiation. *Geophys. Res. Lett.* 2021; **48**: e2021GL095299.
32. Wang L, Dai L and Gong W *et al.* Subduction initiation at the Solomon Back-Arc Basin: Contributions from both island arc rheological strength and oceanic plateau collision. *Geophys. Res. Lett.* 2022; **49**: e2021GL093369.
33. García-Reyes A and Dyment J. Structure, age, and origin of the Caribbean Plate unraveled. *Earth Planet. Sci. Lett.* 2021; **571**: 117100.
34. Dunn RA and Hernandez O. Tracking blue whales in the eastern tropical Pacific with an ocean-bottom seismometer and hydrophone array. *J. Acoust. Soc. Am*. 2009; **126**: 1084–1094.
35. Dunn RA, Lekić V and Detrick RS *et al.* Three-dimensional seismic structure of the Mid-Atlantic Ridge (35°N): Evidence for focused melt supply and lower crustal dike injection. *J. Geophys. Res. Solid Earth* 2005; **110**: B09101.
36. Grevemeyer I, Ranero CR, and Ivandic M. Structure of oceanic crust and serpentinization at subduction trenches. *Geosphere* 2018; **14**: 395–418.
